# The emotional perception and cognitive processing during multimedia learning in students with depressive tendencies

**DOI:** 10.3389/fpsyg.2025.1674489

**Published:** 2025-09-10

**Authors:** Ziyi Wang, Junfei Wang, Chengwei Liu, Huiying Liu, Lei Cui

**Affiliations:** ^1^Faculty of Psychology, Shandong Normal University, Jinan, China; ^2^Shandong Provincial Key Laboratory of Brain Science and Mental Health, Jinan, China

**Keywords:** emotional perception, cognitive processing, visual channel, auditory channel, students with depressive tendencies, eye-tracking technology

## Abstract

The impact of emotional design of pedagogical agent on learners' emotional perception and cognitive processing is the research focus in multimedia learning studies. While pedagogical agent's positive emotion facilitates learning among healthy students, how the pedagogical agent's emotion influences the emotional perception and cognitive processing in students with depressive tendencies remains unclear. This study investigated this issue by presenting emotional cues through both visual (facial expression) and auditory (voice tone) channels to students with depressive tendencies. Results found that learners perceived the negative emotion but not the positive emotion conveyed by the pedagogical agent's facial expression, supporting Beck's cognitive theory of depression. Compared to negative voice tone, positive voice tone enhanced the social connection between learners and the pedagogical agent, aligning with the positivity principle and emotional contagion theory. In addition, when the pedagogical agent's emotions across visual and auditory channels was congruent (both positive or negative), learners exhibited increased germane cognitive load and improved performance on transfer tests, supporting the congruency effect. Therefore, for learners with depressive tendencies, multimedia designers should prioritize auditory emotional cue and utilize the emotional congruency effect across channels to facilitate learner's emotional perception and cognitive processing.

## 1 Introduction

Depression has become a mental health issue of global concern. Data from the World Health Organization (WHO) shows that depression accounts for up to 10% of the global burden of non-fatal diseases (World Health Organization, 2016, p. 4), which significantly increases the disease burden of the whole world (Shim et al., 2012). As of 2023, approximately 280 million people worldwide suffer from depression, with over 700,000 deaths by suicide each year, and suicide is the fourth leading cause of death among individuals aged 15 to 29 (World Health Organization, 2016). Notably, a meta-analysis study shows that the incidence rate of depressive symptoms among university students worldwide is as high as 33.6% (Li et al., 2022; Kessler et al., 2003). In China, data from the *2023 China Mental Health Blue Book* shows that the detection rate of depression risk among university students is 24.18%. Depression not only has a negative impact on students' emotional and psychological states, but also impairs cognitive functions, thereby interfering with learning and daily life (Hysenbegasi et al., 2005; Gotlib et al., 2014). The manner to improve the learning efficiency of university students with depressive tendencies is an important and practically significant research question. However, research in this area is scarce, and requires further exploration.

The rapid development of information technology promotes the popularization of multimedia learning, where educators present information through multiple media forms (Mayer and Mayer, 2005). Multimedia learning emphasizes presenting learning information through visual and auditory channels simultaneously, thereby enabling learners to use different sensory channels for learning and form a multi-channel complementary learning advantage. As widely recognized, the teacher–student interaction during learning can enhance students' learning motivation and effectiveness, however, multimedia learning lacks teacher–student interaction (Wang et al., 2023). To this concern, researchers add pedagogical agent to realize teacher–student interaction and aim to enhance learners' motivation and the capability to process information (Kang et al., 2020; Heng et al., 2025). In recent years, more and more researchers have found that the emotion of pedagogical agent can affect the effectiveness of multimedia learning, which shows that compared with pedagogical agent with neutral or negative emotion, those with positive emotion significantly enhance learners' positive emotion and intrinsic motivation, and thereby promoting learning performance (Ba et al., 2021; Lawson et al., 2021a,b,c; Liew et al., 2017). However, most existing studies have been conducted with healthy students, seldom was known about the students with depressive tendencies. Given the high incidence of depressive tendencies among students and the unique emotional perception pattern of them, exploring multimedia learning in students with depressive tendencies has significant theoretical and practical importance.

### 1.1 Pedagogical agent's positive emotion facilitates multimedia learning

Multimedia learning presents information through multiple media forms, such as text (oral/written) and image (pictures/videos), with advantages of including but not limited, multi-channel complementary synergy, efficient and intuitive information presentation, and flexible learning methods (Mayer and Mayer, 2005). As widely recognized, the teacher–student interaction during learning could enhance students' learning motivation and effectiveness, however, traditional multimedia learning does not include teacher–student interaction (Wang et al., 2023). To address this limitation, researchers incorporate pedagogical agent to compensate for the absence of face–to–face communication between learners and teachers in multimedia learning, for the purpose to enhance social connections and emotional support during the learning, thereby improving learners' initiative and emotional experience, and promoting learning outcomes (Bower, 2019; Lee et al., 2018). A pedagogical agent is an on-screen character, which could be a virtual image or a real person image, designed to support learners' by delivering instructional information (Heidig and Clarebout, 2011; Beege et al., 2020).

The influence of emotional design of pedagogical agent on multimedia learning is a research hotspot. Liew et al. (2017) designed two pedagogical agents with different emotional expressions in a computer programming course, an enthusiastic pedagogical agent and a neutral pedagogical agent. The results showed that learners in the enthusiastic agent group had significantly higher scores for positive emotional perception, intrinsic motivation, retention, and comprehension, compared with learners in the neutral agent group. Similarly, Ba et al. (2021) revealed that learners with the positive pedagogical agent had higher pride emotion and higher transfer test scores compared to the neutral pedagogical agent condition. Guo and Goh (2016) examined how pedagogical agent's emotional expression affects learning in an online information literacy education tutorial, and the results showed that compared to neutral pedagogical agent and no-agent conditions, positive pedagogical agent significantly improved participants' affective enjoyment, usage intention, and motivation. In summary, existing studies indicate that compared with pedagogical agent with neutral emotion, those with positive emotion could make learners recognize and perceive similar emotion, thereby enhance social connections between learners and pedagogical agent, and improve learners' motivation, engagement, and learning performance. Besides, there were some studies compared the effects of positive pedagogical agent (such as happy/content) with negative pedagogical agent (such as boring/frustrated) to explore the effect of positive pedagogical agent. The results showed that compared with negative pedagogical agent, positive ones enhanced learners' learning motivation and engagement (Horovitz and Mayer, 2021; Lawson et al., 2021a,b), and improved learners' performance on subsequent tests (Lawson et al., 2021c).

Collectively, these studies indicate that pedagogical agent with positive emotion could significantly improve learners' emotional state, motivation, engagement, and learning performance. The above empirical research in multimedia learning supported the positivity principle (Lawson et al., 2021b; Plass et al., 2020), that is, when pedagogical agent shows positive emotion in the teaching processing, learners perceive the positive emotion and establish better relationship with pedagogical agent, thereby, improve learners' motivation and ultimately achieve better learning outcomes. Correspondingly, the results also strongly support the Emotional Contagion Theory (Hatfield et al., 1993; Frenzel et al., 2021), which hold that an individual's emotional state is automatically and unconsciously affected by others' emotion, emphasizes the “infectious” characteristic of emotion in interpersonal interaction. When people perceive others' emotion, they often unconsciously imitate this emotional expression, thereby affecting their own emotional perception and behavioral performance (Niedenthal, 2007).

To sum up, in the context of multimedia learning, the positive emotion of pedagogical agent promotes learners' emotional resonance and make them generate positive emotional perception, thereby enhance learners' trust and dependence on pedagogical agent, strengthens their learning motivation, and ultimately promotes learning effectiveness. However, the current research has been conducted exclusively with healthy students. It remains unexplored whether these positive effects are also applicable to students with depressive tendencies.

### 1.2 The presenting channel of pedagogical agent's emotion cue and multimedia learning

The pedagogical agent's emotion cues are usually presented through visual or/and auditory channels (Keltner et al., 2019). The core cues of the visual channel and the auditory channel are facial expression and voice tone, respectively (Ba et al., 2021; Lester et al., 1998).

The facial expression of pedagogical agent refers to the movements of the facial muscles of pedagogical agent during teaching to express their emotion. Research hypothesized that positive emotion conveyed by the facial expression of pedagogical agent could affect students' learning motivation, attention, and then promote learning (Frenzel et al., 2021; Pi et al., 2023; Wang, 2022). Correspondingly, several studies proved that positive facial expression of pedagogical agent could effectively stimulate students' learning motivation compared to neutral or negative ones, thereby improving learning performance (Horovitz and Mayer, 2021; Pi et al., 2022, 2023). Moreover, some studies showed that the positive facial expression of pedagogical agent was positively correlated with learning performance, while negative facial expression was negatively correlated with learning performance (Beege et al., 2018; Horan et al., 2012; Pi et al., 2023).

The voice tone refers to the voice prosody in learning videos that showing pedagogical agent's emotion, which plays a crucial role in students' learning. According to Liew et al. (2017), humans could perceive emotion from speakers' voice, which evoked related emotion accordingly. Similarly, for multimedia learning, emotional expressing by pedagogical agent through intonation causes different emotional reactions from learners. This view had been widely confirmed by subsequent studies that found positive intonation can arouse learners' sense of closeness, enhance their learning motivation, and thereby improve their learning performance (Keller et al., 2016; Davis et al., 2019; Lawson and Mayer, 2022).

The pedagogical agent's emotion is usually presented through both visual and auditory channels simultaneously, and the human multi-sensory integration processing plays the most important role during emotional perception (Zhang et al., 2024), thus research and theories considering both channels has more practical significance. According to Mehrabian's rule, regarding the channel proportion in expression transmission during daily interpersonal communication, it involves 7% of verbal expression, 38% of vocal expression, and 55% of facial expression (Mehrabian, 1971). However, it is unclear whether the transmissions of facial expression and voice emotion of pedagogical agent in multimedia learning follow this rule, which deserves further research. Moreover, according to Mayer's modality principle, which emphasizes that learning is optimized when auditory explanations accompanying visual materials through dual-channel processing (Mayer and Mayer, 2005; Mayer, 2008). Existing multimedia learning studies mainly focused on the overall role of synchronously presented emotional cues of pedagogical agent, which may be presented through visual and auditory dual-channel. Nevertheless, the different role of emotional cues between visual and auditory channels needed to be analyzed; furthermore, the combined role of dual-channel emotional cues, also needed to be explored, which was the purpose of present study. According to the congruency effect, the congruent emotion conveyed by different emotional cues of pedagogical agent (e.g., facial expressions and body movements) has a positive impact on an individual's emotional perception (Abramson et al., 2021; Huestegge et al., 2019; Pi et al., 2023). Consequently, we hypothesized that when facial expression and voice tone of pedagogical agent are presented together, congruent facial and vocal expressions will enhance learning more than incongruent expressions.

### 1.3 Pedagogical agent's emotion and emotional perception in students with depressive tendencies

Depressive tendencies refer to a state of subthreshold depressive symptoms where individuals exhibit clinically relevant manifestations without meeting the full diagnostic criteria for major depressive disorder (Kroenke et al., 2001). These individuals share cognitive characteristics with clinically diagnosed depression patients. As a risk factor for major depressive disorder, depressive tendencies not only impair quality of life, but also disrupt learning processes through specific emotional perception (Zhang et al., 2019).

According to Beck's cognitive theory of depression, individuals with depression exhibit negative attentional bias and impaired positive emotion recognition compared with healthy individuals (Beck, 2002). Negative attentional bias refers to the tendency to preferentially attend to negative emotional information in emotional experiences. This manifest as sustained attention toward negative stimuli with difficulty in disengagement, which promote prioritized processing of negative external information while attenuate allocation to positive or neutral stimuli (Kovacs and Beck, 1978). Concurrently, impaired positive emotion recognition reflects disruptions in both encoding and decoding of positive affective cues, leading to the misinterpretation of positive emotion as neutral or negative (Mikhailova et al., 1996; Demiralp et al., 2012). Regarding visually emotional processing, Kellough et al. (2008) found that depressed groups exhibited more time viewing negative images and less time viewing positive images compared with healthy controls. This pattern of hyper-attention to negative information and neglect of positive information results in barriers to information processing during learning, thereby compromising learning outcomes. Bourke et al. (2010) demonstrated that depressed individuals more readily interpreted positive facial expression as negative, impairing their ability to accurately detect subtle changes in others' facial expression. Naranjo et al. (2011) further revealed a tendency among depressed patients to misclassify faces with neutral emotion as expressing negative emotion. In auditory emotion processing, Kan et al. (2004) observed that depressed patients misinterpreted neutral prosody as conveying negative emotion. Notably, Naranjo et al. (2011) found no significant differences between depressed and healthy control groups in recognizing positive emotion conveyed by music. In summary, depressed individuals demonstrate distinct patterns of emotional processing compared with the health population in both visual and auditory channels, with the possibility of cross-channel. However, these findings require further evidence.

As analyzed above, students with depressive tendencies have different emotional perception pattern compared with the healthy counterparts, which should affect the perception of emotional information conveyed by pedagogical agent and further influence multimedia learning. Accordingly, the present study explored the emotional perception and cognitive processing in multimedia learning among students with depressive tendencies; examined the effect of emotional cue through visual or auditory channel and the emotional congruency effect of across channels. This research could help illuminate the unique cognitive mechanisms in depression tendency learners during multimedia learning, and provide empirical guidance for learning designers to ultimately enhance learning outcomes for this population in digital learning environments.

### 1.4 Application of eye-tracking technology in multimedia learning research

Eye-tracking technology records learners' eye movement trajectories in real time, captures time-varying cognitive processes during learning activities and links learning outcomes with internal cognitive mechanisms (Zheng et al., 2023). Compared to traditional methods, this technology provides immediate and objective data for in-depth analysis of learners' attentional allocation when processing learning materials (Rayner, 1998). Consequently, eye-tracking technology is being applied more and more widely in multimedia learning research.

In research on pedagogical agent, eye-tracking metrics are valuable in revealing attentional patterns. Studies indicate that learners' fixation time on learning materials typically reflects the depth of information processing (Li et al., 2016; Pi et al., 2023). For instance, Li et al. (2016) found that incorporating pedagogical agent increased learners' fixation time on core learning content compared with no-agent conditions, suggesting that pedagogical agent did not distract attention from essential materials, but might instead enhancing learning outcomes. Eye-tracking technology further provides refined metrics for examining attentional allocation pattern. The proportion of fixation time within Areas of Interest (AOIs) refers to the ratio of time spent fixating on specific regions to total viewing time during multimedia learning (Alemdag and Cagiltay, 2018). The number of transitions quantifies attention shifts between distinct AOIs, indicating attentional dispersion or cognitive coordination (Pi et al., 2023).

In summary, eye-tracking technology captures learners' attentional allocation objectively during multimedia learning. To the best of our knowledge, previous eye-tracking research in multimedia learning focused primarily on healthy university students, this is the first study on university learners with depressive tendencies. This study was designed to integrate post-learning subjective questionnaire data with real-time processing patterns reflected in eye movements to comprehensively understand learners' cognitive processing across different processing stages.

### 1.5 The present study

This study aimed to investigate the effects of pedagogical agent's emotional expression on multimedia learning among students with depressive tendencies. The specific objectives include: (1) to examine the impact of emotional cues in visual and auditory channels on multimedia learning; (2) to explore how the emotional congruency across channels affect multimedia learning. The present study manipulated two independent variables: facial expression (happy vs. boring) and voice tone (happy vs. boring). Multiple dependent variables were measured, including emotion perception, motivation, transfer test performance, cognitive load (extraneous, intrinsic, germane), social connection, and eye-tracking metrics, such as AOI fixation time, fixation time ratio, and AOI transition counts.

Firstly, based on Beck's cognitive theory of depression, we hypothesized that for individuals with depressive tendencies, positive facial expression was not expected to significantly alter learners' emotional perception and learning efficiency. Secondly, according to the study of Naranjo et al. (2011), positive voice tone would enhance emotional perception, cognitive processing and learning efficiency. Third, according to congruency effect (Abramson et al., 2021; Huestegge et al., 2019; Pi et al., 2023), when the emotions across visual and auditory channels were congruent, no matter both positive or both negative, it would facilitate learning performance.

## 2 Methods

### 2.1 Participants

The required sample size for this study was calculated using G^*^Power software. With a fixed effect size (*f* ) of 0.45, a statistical power of 0.80, and a significance level (α) of 0.05, the calculation yielded a required sample size of 41.

Prior to the experiment, the Beck Depression Inventory (BDI; Beck et al., 1961) was administered online to students at a university in China. This self-report scale, originally developed by Beck, consists of 21 items, each rated on a 0-to-3-point scale, with higher total scores indicating higher levels of depression. The Chinese version translated by Wang et al. (2011) was used in this study. According to the diagnostic criteria established by Xu (1991), participants scoring 10 or above were suspected to have depression, indicating at least some degree of emotional disturbance.

According to the sample size requirements, fifty-nine university students whose BDI scores were 10 or above were selected as participants. Two of them were excluded due to excessive eye movement drift, and another one was excluded for failing to complete the transfer test and the social connection scale. This resulted in 56 valid participants (37 females), aged 19 to 25 years (*M* = 21.23, *SD* = 1.70). All participants were randomly assigned to one of four experimental conditions. Participants were unaware of the experimental purpose prior to the experiment and received monetary compensation upon completion. Additionally, none of the participants reported color blindness, color vision deficiencies, or dyslexia, and all had normal or corrected-to-normal vision. This study was approved by the Ethics Committee of Shandong Normal University.

### 2.2 Experimental design

This study employed a 2(facial expression: happy vs. boring) × 2(voice tone: happy vs. boring) between-subjects factorial design. The dependent variables included recognition of the agent's emotions, the learner's experienced emotions, transfer test performance, intrinsic motivation, cognitive load (extraneous, intrinsic, and germane), social connection, and eye-tracking metrics (fixation duration in areas of interest, fixation duration ratio in areas of interest, and number of transitions between areas of interest). Control variables were prior knowledge level and emotional state before experiment.

Collectively, these indicators measure how the pedagogical agent's emotions influence the learning processes and outcomes for students with depressive tendencies. Emotion and motivation reflect the learner's affective state and willingness to engage, serving as the driving forces behind learning. The transfer test directly gauges learning effectiveness by evaluating the flexible application of knowledge. Cognitive load assesses the mental burden of processing information, with the ideal being to reduce irrelevant burdens (extraneous and intrinsic) while increasing beneficial investment (germane). Social connection, reflecting the learner's sense of closeness and trust toward the agent, which could enhance learning motivation.

### 2.3 Experimental materials

The learning materials were generated according to “The Principles of Lightning Formation” (Wang et al., 2015; Mayer and Mayer, 2005; Li et al., 2020; Zhao and Mayer, 2025). Four instructional videos featuring a pedagogical agent were developed, each covering the same topic. Following the methodologies of Mayer and Mayer (2005) and Wang et al. (2015), the videos presented “The Principles of Lightning Formation”, and a virtual pedagogical agent was incorporated. In all four videos, a female pedagogical agent appeared on the right side of the slides. The agent's facial expressions and voice were present throughout the entire duration of each video. The study utilized four distinct learning videos, except for the emotional valence of the facial expressions and voice, all other contents were identical across the videos. The two videos with happy voice condition lasted for 1 min and 55 s, while the two videos with boring voice condition lasted for 2 min and 27 s. The spatial arrangement of the learning materials and the pedagogical agent within the video frame is illustrated in **Figure 2**.

Referring to the emotional manipulations of pedagogical agents in the studies by Lawson et al. (2021a,b,c) and Liew et al. (2017), the happy facial expression was designed to feature a constant smile, with raised corners of the mouth, and pronounced eyebrow movements during speech. In contrast, the boring facial expression involved a flat mouth, a slightly parted mouth, occasional pursing of the lips, and occasional frowning. The happy voice of the pedagogical agent was designed to be optimistic, energetic, with large variations in pitch and tone, and fast speaking speed. The boring voice was monotonous and flat, exhibiting minimal changes in pitch and tone, and slow speaking speed.

### 2.4 Experimental procedure

**Figure 1:**
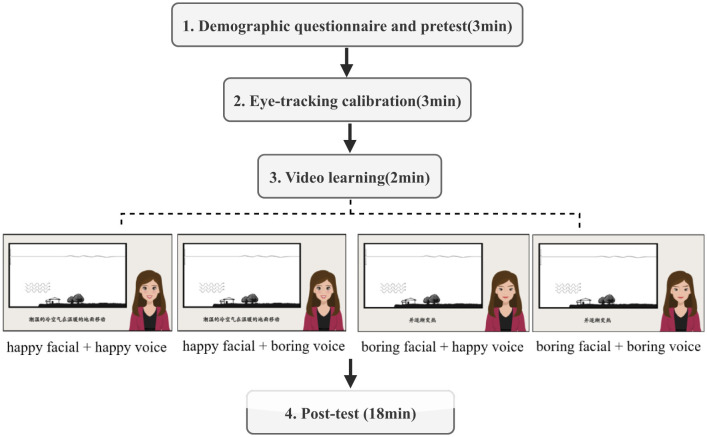
The experimental procedure of the study, which included four stages lasting for approximately 26 min.

The experimental procedure is illustrated in Figure 1, which included four stages:

(1) First, participants completed demographic information, followed by a prior knowledge test and a pre-test of emotion assessment.
(2) Second, participants were invited into the eye-tracking laboratory for seat adjustment. They then received experimental instructions. Subsequently, participants were seated in front of the eye tracker with their chin placed on a chin rest, and a 9-point calibration was performed to ensure accurate recording of eye movement trajectories.
(3) Third, participants were randomly assigned to one of the four experimental groups to watch corresponding videos, during which their eye movements were tracked.
(4) Finally, immediately after watching the video, participants completed a post-test of emotions assessment, followed by questionnaires assessing recognition of the pedagogical agent's emotions, a motivation scale, a cognitive load scale, a learning transfer test, a social connection questionnaire, and a cognitive and affective processing questionnaire. The entire experiment lasted ~26 min. Upon completion of the experiment, participants received corresponding compensation.

### 2.5 Eye-Tracking data collection and analysis

This study employed the Eye-link 1,000 Plus eye tracker, produced by SR Research, Canada, with a sampling rate of 1,000 Hz. The learning videos were presented on a 21-inch computer monitor (Zhang et al., 2018), and the audio was played through headphones. Participants' eyes were positioned 60 cm away from the screen, with a screen resolution of 1,024 × 768 pixels and a refresh rate of 160 Hz. During the experiment, the eye movement trajectory of each participant's right eye was recorded.

**Figure 2:**
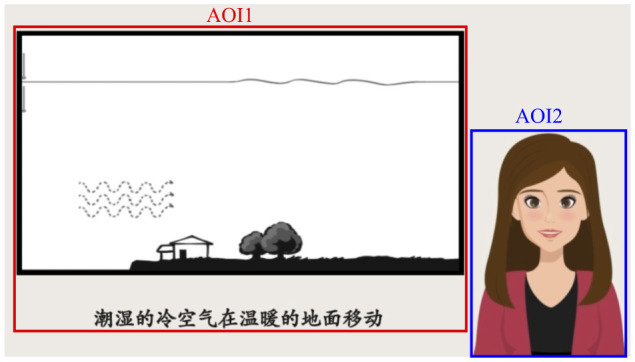
The two areas of interest (AOIs) in the video learning stage. The left AOI1 was for Learning Material; the right AOI2 was for Pedagogical Agent.

We designated two Areas of Interest (AOIs): the learning material area (AOI1) and the pedagogical agent area (AOI2) (see Figure 2). The indicators selected for analysis included fixation time on the AOIs, fixation time ratio on the AOIs, and the number of transitions between AOIs. Fixation time refers to the amount of time focusing on an AOI during the learning process. According to the Eye-Mind Hypothesis (Eye-Mind Assumption, Just and Carpenter, 1980), the amount of time spent fixating on specific information reflects the cognitive processing time of that information. The longer fixation time, the more attention and cognitive processing are directed toward that AOI (Dirkx et al., 2021; Li et al., 2016; Wang et al., 2015). The fixation time ratio is the ratio of the fixation time to the total observation time and serves as indicator for understanding how learners distribute their attention among different content elements during multimedia learning (Alemdag and Cagiltay, 2018; Park et al., 2020). A higher fixation time ratio indicates that participants spent more time observing that AOI relative to the others. The number of transitions refers to how many times participants shift their gaze between the AOIs, a higher number of transitions between the learning material and the pedagogical agent indicates more integration between the different AOI (Pi et al., 2023).

### 2.6 Measurement tools

The subjective measurements in this study included emotions, motivation, transfer test performance, cognitive load (extraneous, intrinsic, and germane), and social connection. The definitions and measurement tools of each dependent variable are as follows:

(1) **Prior knowledge:** primarily used to assess participants' pre-existing knowledge of the relevant learning topic. The questionnaire was developed by Harp and Mayer (1997) and later revised by Feng (2020). It contains 4 items on meteorological knowledge (e.g., “I know how lightning is formed.”), rated on a 5-point scale, with a maximum total score of 20 points.
(2) **Positive emotions:** These refer to the positive emotions perceived by learners after learning (such as being active, happy, energetic, etc.). The positive emotion questionnaire was selected from the Positive and Negative Affect Schedule (PANAS, Watson et al., 1988), revised by Qiu et al. (2008). An example question is, “I feel happy.” It uses a 5-point Likert scale ranging from 1 (completely disagree) to 5 (completely agree). The final score is the sum of each item score. A higher score on the Positive Affect Scale indicates stronger positive emotions.
(3) **Motivation:** motivation refers to the intrinsic drive shown by learners throughout the learning process (Isen and Reeve, 2005).
  The motivation questionnaire was developed by Isen and Reeve (2005) and contains 8 items. It employs a 7-point Likert scale (e.g., “It made me want to explore it further”), where 1 representing strongly disagree and 7 representing strongly agree. The final analysis utilized the average score across the 8 items. A higher score indicates higher intrinsic motivation. This scale has been widely used in previous studies in the multimedia field (Plass et al., 2014; Um et al., 2012; Gong et al., 2017; Xue et al., 2015). In Xue et al. (2015) study, the internal consistency coefficient (Cronbach's alpha) of the motivation scale was 0.93.
(4) **Cognitive load:** Cognitive load refers to the degree of cognitive resource consumption experienced by learners when completing learning tasks. Extraneous cognitive load is caused by information processing irrelevant to learning, such as the design of learning materials; intrinsic cognitive load is determined by the complexity of the learning materials themselves and is related to the learner's individual knowledge level; germane cognitive load includes all processes required for understanding information and constructing schemas (Sweller et al., 2011).
  The cognitive load scale was developed by Leppink et al. (2013) and revised by Xiong et al. (2018). It consists of 13 items rated on an 11-point scale, covering three dimensions, extraneous cognitive load (4 items; e.g., “The explanations and descriptions in the video are very unclear.”), intrinsic cognitive load (4 items; e.g., “The content of the video is very complex.”), and germane cognitive load (5 items; e.g., “In this study, I invested a lot of mental effort to enhance my knowledge and understanding.”). The final score is the average for each dimension. A higher score indicates a higher level of cognitive load in that dimension. In Xiong et al. (2018) study, the reliability coefficients (Cronbach's alpha) for the dimensions were 0.74, 0.82, and 0.91, respectively.
(5) **Transfer test:** The transfer test was revised based on the research of Shangguan (2017) and Feng (2020), consisting of 4 short-answer questions (e.g., “How can the intensity of lightning be reduced?”). Each question contains two scoring points and thus its maximal score is two, thus the maximal score for the test is 8. Participant's answer and score were evaluated by two graduate students whom was rigorously trained, and the final score is the average of the two scorers' rating. The inter-rater reliability coefficient was 0.90.
(6) **Social connection:** it refers to the sense of interpersonal interaction felt by learners with the pedagogical agent during the learning process (Baylor and Ryu, 2003). The social connection questionnaire was adapted from the Agent Persona Instrument (API; Baylor and Ryu, 2003). The scale includes 10 items assessing the extent to which the pedagogical agent facilitates learning (e.g., “The pedagogical agent made me think more deeply about the learning materials.”), 4 items evaluating the agent's credibility (e.g., “The pedagogical agent is knowledgeable.”), 4 items evaluating the agent's human-likeness (e.g., “The pedagogical agent is human-like.”), and 5 items evaluating the agent's engagement (e.g., “The pedagogical agent is motivating.”). The final score was the average of the four dimensions.

## 3 Results

### 3.1 Descriptive results of subjective measurements

Table 1 presents the descriptive statistical results of all subjective measurement variables.

**Table 1:** . Means (*M*) and standard deviations (*SD*) of all pre-test and post-test variables across the four conditions.

**Dependent variable**	**Happy-facial expression**		**Boring-facial expression**	
	**Happy-voice tone**	**Boring-voice tone**	**Happy-voice tone**	**Boring-voice tone**
**Pre-test**				
Depression score	13.71 (4.10)	15.79 (5.04)	15.08 (3.62)	13.73 (1.34)
Prior knowledge	10.07 (4.89)	12.00 (4.76)	10.54 (4.12)	10.73 (5.44)
PANAS				
PA	32.71 (5.22)	29.29 (4.66)	31.59 (3.93)	33.20 (4.06)
NA	13.93 (4.63)	16.14 (6.27)	13.95 (5.59)	13.60 (3.56)
**Post-test**				
Emotion perception				
Happy rating of agent	3.50 (1.02)	1.29 (0.47)	3.15 (1.07)	1.80 (1.08)
Boring rating of agent	2.50 (1.35)	3.36 (0.75)	4.27 (0.80)	3.23 (1.17)
Emotion	29.86 (6.10)	22.57 (7.34)	29.00 (8.52)	27.60 (6.38)
Motivation	4.71 (1.58)	3.71 (1.45)	4.30 (1.32)	4.54 (1.55)
Cognitive load				
Extraneous cognitive load	3.82 (2.04)	4.35 (1.14)	3.98 (2.10)	3.73 (2.66)
Intrinsic cognitive load	6.18 (1.97)	5.48 (2.44)	5.71 (2.36)	5.33 (2.11)
Germane cognitive load	7.46 (1.09)	6.52 (1.32)	6.43 (1.38)	7.68 (0.97)
Social connection				
Facilitated learning	2.89 (0.69)	1.96 (0.68)	2.83 (0.52)	2.39 (0.51)
Credible	3.29 (0.49)	2.56 (0.78)	3.28 (0.48)	2.87 (0.68)
Human-likeness	3.01 (0.49)	2.94 (0.66)	3.03 (0.76)	2.96 (0.31)
Engagement	3.50 (0.62)	3.31 (0.61)	3.38 (0.87)	3.36 (0.74)
Learning performance				
Transfer test	3.29 (1.49)	2.43 (1.60)	3.08 (0.95)	3.73 (0.96)

Standard deviations are in parentheses. PA, Positive Affect; NA, Negative Affect.

To ensure that the experimental effects were not caused by prior knowledge and emotional states, we first conducted a one-way analysis of variance (ANOVA) on the prior knowledge test scores across the four conditions. The results showed no significant difference in prior knowledge levels, pre-test positive and negative emotions among the experimental groups, *Fs* < 2.14. The means and standard deviations of each pre-test variable are shown in Table 1.

### 3.2 Emotional perception

A 2 (facial expression: happy, boring) × 2 (voice: happy, boring) between—subjects analysis of variance (ANOVA) was conducted with post-test variables. Regarding learners' recognition of the pedagogical agent's emotions, in the evaluation of the agent's happy emotion, the main effect of voice was significant, with higher scores in the happy voice condition compared with the boring voice condition, *F*_(1, 55)_ = 49.76, *p* < 0.001, η^2^ = 0.49; the main effect of facial expression and the interaction between facial expression and voice were not significant, *Fs* < 2.89. In the evaluation of the pedagogical agent's boring emotion, the main effect of facial expression was significant, with higher scores in the boring facial expression condition compared with the happy facial expression condition, *F*_(1, 55)_ = 8.73, *p* = 0.005, η^2^ = 0.14; the main effect of voice was significant, with higher scores in the boring voice condition compared with the happy voice condition, *F*_(1, 55)_ = 11.63, *p* = 0.001, η^2^ = 0.18; the interaction between facial expression and voice was not significant, *F* = 0.10.

For learners' positive emotions, the main effect of voice was significant, with higher scores in the happy voice condition compared with the boring voice condition, *F*_(1, 55)_ = 5.22, *p* = 0.026, η^2^ = 0.09; the main effect of facial expression and the interaction between facial expression and voice were not significant, *Fs* < 2.40.

### 3.3 Learning performance

**Figure 3:**
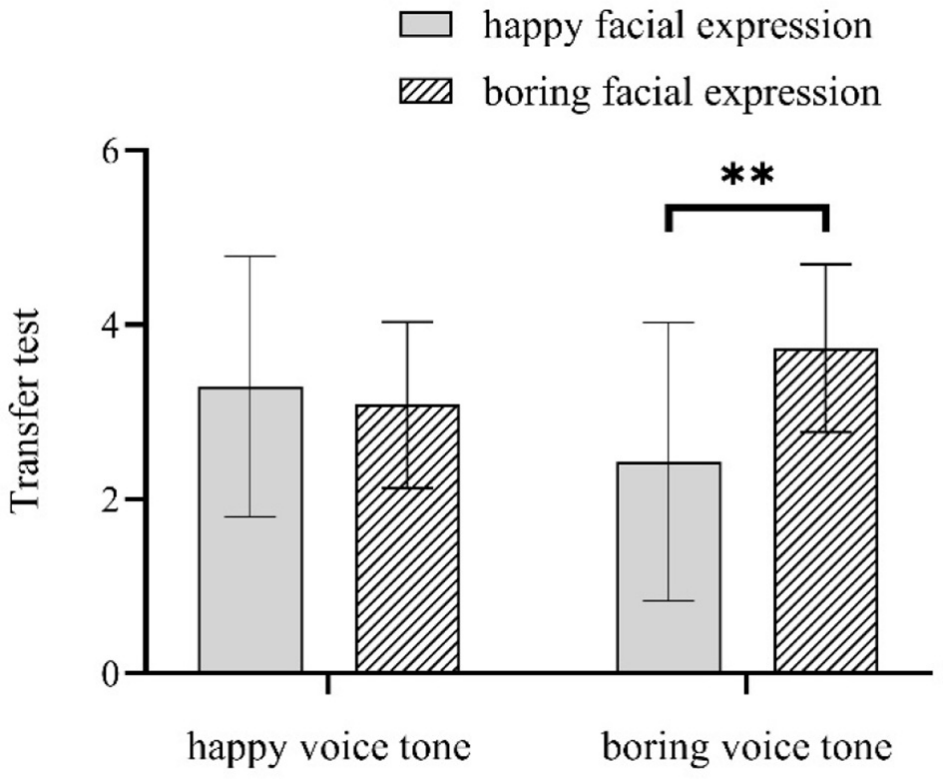
Interaction between facial expression and voice tone in the transfer test (** means *p* < 0.01).

For the transfer test, the main effects of facial expression and voice were not significant, *Fs* < 2.53; the interaction between facial expression and voice was significant, *F*_(1, 55)_ = 4.83, *p* = 0.032, η^2^ = 0.09. In the happy voice condition, there was no significant difference between the happy facial expression condition and the boring facial expression condition, *F* = 0.18; in the boring voice condition, the transfer test score for the boring facial expression group was higher than the happy facial expression group, *F*_(1, 55)_ = 7.44, *p* = 0.009, η^2^ = 0.13. Figure 3 shows the interaction effect.

### 3.4 Cognitive processing

Regarding motivation, extraneous cognitive load, and intrinsic cognitive load, the main effects of facial expression and voice, and the interaction between facial expression and voice were not significant, *Fs* < 2.46.

**Figure 4:**
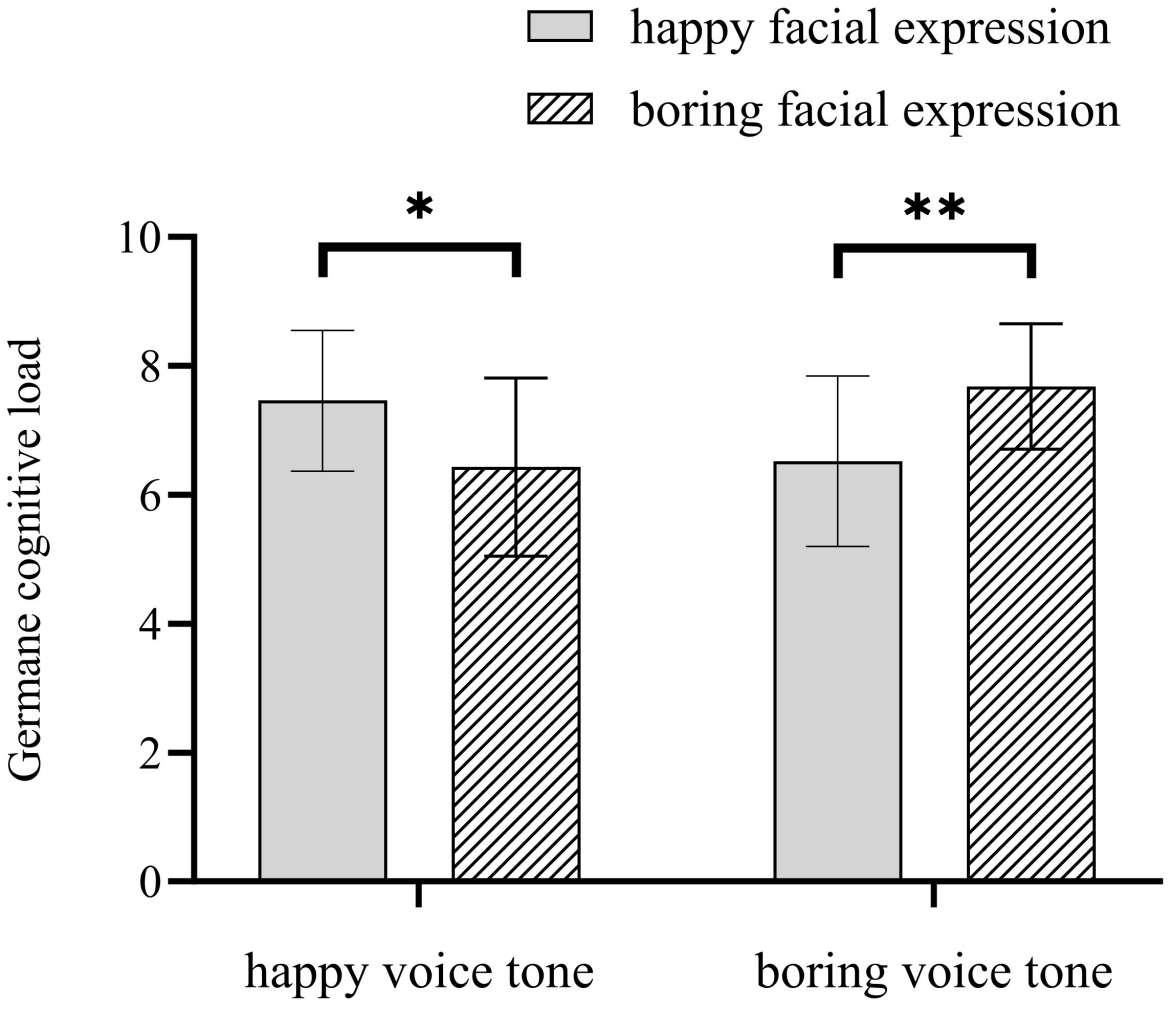
Interaction between facial expression and voice tone in the germane cognitive load (* means *p* < 0.05; ** means *p* < 0.01).

For germane cognitive load, the main effects of facial expression and voice were not significant, *Fs* < 0.24; the interaction between facial expression and voice was significant, *F*_(1, 55)_ = 11.66, *p* = 0.001, η^2^ = 0.18. In the happy voice condition, the germane cognitive load score for the happy facial expression condition was higher than the boring facial expression condition, *F*_(1, 55)_ = 4.99, *p* = 0.030, η^2^ = 0.09; in the boring voice condition, the boring facial expression group was higher than the happy facial expression group, *F*_(1, 55)_ = 6.78, *p* = 0.001, η^2^ = 0.12. Figure 4 shows the interaction effect.

### 3.5 Social connection

For “Facilitative learning”, the main effect of voice was significant, with higher scores in the happy voice condition compared with the boring voice condition, *F*_(1, 55)_ = 18.02, *p* < 0.001, η^2^ = 0.26. The main effect of facial expression and the interaction between facial expression and voice were not significant, *Fs* < 2.24.

For “Credibility”, the main effect of voice was significant, with higher scores in the happy voice condition compared with the boring voice condition, *F*_(1, 55)_ = 11.56, *p* = 0.001, η^2^ = 0.18. The main effect of facial expression and the interaction between facial expression and voice were not significant, *Fs* < 0.91.

For “Human-likeness” and “Engagement”, the main effects of facial expression and voice, as well as the interaction between facial expression and voice were not significant, *Fs* < 1.

### 3.6 Eye movement indicators

According to the standards of eye movement tracking quality control and extreme data processing, data cleaning was carried out according to the following standards:

(1) Eye movement data tracking was lost or there was a large-amplitude drift in eye movement, and data of 2 participants were deleted;
(2) After testing the normality of the fixation time in the area of interest of participants, the data of the fixation time in the area of interest outside ±2.5 standard deviations were eliminated. The eliminated data accounted for about 3.1% of the total data. The means and standard deviations of eye movement indicators are shown in Table 2. A 2 (facial expression: happy, boring) × 2 (voice: happy, boring) between—subjects analysis of variance (ANOVA) was conducted on eye movement indicators (fixation time, fixation time, and number of transitions).

**Table 2:** Means (*M*) and standard deviations (*SD*) of all eye-tracking variables across the four video lectures.

**Dependent variable**	**Happy-facial expression**		**Boring-facial expression**	
	**Happy-voice tone**	**Boring-voice tone**	**Happy-voice tone**	**Boring-voice tone**
**Fixation time (ms)**				
Learning material	87,366 (9,120)	1,12,380 (12,457)	93,754 (6,906)	1,14,626 (1,2,930)
Pedagogical agent	3,085 (2,034)	3,930 (1,926)	2,971 (2,891)	3737 (2,378)
**Ratio of fixation time**				
Learning material	0.93 (0.05)	0.93 (0.05)	0.96 (0.03)	0.95 (0.04)
Pedagogical agent	0.03 (0.02)	0.04 (0.02)	0.03 (0.03)	0.04 (0.03)
**Transitions**	16.93 (12.68)	24.43 (8.60)	16.65 (12.06)	21.98 (10.89)

Standard deviations are in parentheses.

For the fixation time on learning materials, the main effect of voice was significant, with the boring voice condition leading to longer fixation times compared with the happy voice condition, *F*_(1, 55)_ = 63.58, *p* < 0.001, η^2^ = 0.55; the main effect of facial expression and the interaction between facial expression and voice were not significant, *Fs* < 2.25.

For the fixation time on the pedagogical agent, the main effects of facial expression and voice, as well as the interaction between facial expression and voice were not significant, *Fs* < 1.68.

For the fixation time ratio on learning materials, the main effects of facial expression and voice, as well as the interaction between facial expression and voice were not significant, *Fs* < 3.92.

For the fixation time ratio on the pedagogical agent, the main effects of facial expression and voice, as well as the interaction between facial expression and voice were not significant, *Fs* < 0.74.

For the number of transitions, the main effect of voice was significant, with the boring voice condition resulting in more transitions than the happy voice condition, *F*_(1, 55)_ = 4.63, *p* = 0.036, η^2^ = 0.08; the main effect of facial expression and the interaction between facial expression and voice were not significant, *Fs* < 0.21.

## 4 Discussion

By combining objective measurements with subjective reports, present study investigated the impact of pedagogical agents' emotion design on emotional perception and cognitive processing among students with depressive tendencies during multimedia learning, and further analyzed the influence of emotional expression through visual or auditory channel as well as the influence of emotional congruency across dual-channel on multimedia learning.

The results showed, when pedagogical agents' emotional expression presented through visual channel cues, the emotional perception of students with depressive tendencies exhibited negative attentional bias and impaired positive emotion recognition. Students could accurately perceive the negative emotional expression of pedagogical agent but failed to perceive the positive emotional expression. Moreover, in terms of cognitive processing and learning outcomes, there was no significant differences between pedagogical agent with negative and positive facial expression. However, when pedagogical agents' emotional expression presented through auditory channel cues, students with depressive tendencies could perceive both positive and negative emotional expression presented by pedagogical agents. Compared with the negative voice tone, the positive voice tone of pedagogical agent significantly enhanced students' social connection with the pedagogical agents, comprising their perception of the pedagogical agent's role in facilitating learning and its credibility.

### 4.1 The impact of emotional expression through the visual channel

The study found that, for the emotion conveyed through facial expression of pedagogical agents, students with depressive tendencies could perceive the negative emotional expression but could not effectively perceive the positive ones. The results were in line with Beck's cognitive theory of depression (Beck, 2002), which states that depressed individuals have high sensitivity and attentional bias to negative emotion perception (Gotlib and Joormann, 2010; Klawohn et al., 2020) and have positive emotional perception impairment concurrently (LeMoult and Gotlib, 2019). More importantly, regardless of whether the facial expression of the pedagogical agent was positive or negative, there was no significant difference in the cognitive processing and learning outcomes, which was contrast to the effects observed in the healthy students.

For the healthy students, research on multimedia learning showed that, firstly, when pedagogical agent expressed positive emotion during the instructional processing, students could effectively perceive the positive emotion, experienced enhanced motivation, and achieved improved learning efficiency, which was called the positivity principle (Horovitz and Mayer, 2021; Pi et al., 2022, 2023; Lawson et al., 2021a,b,c; Plass et al., 2020). Secondly, in terms of learning outcomes, the positive facial expression of pedagogical agent could promote the learning performance, which was congruent with the emotional contagion theory (Frenzel et al., 2021), that is, students were automatically affected by the emotional expression of the agents, thereby enhancing trust in the pedagogical agent and ultimately promoting learning performance (Wang, 2022).

Consequently, we could see clearly that there were significant differences on emotional perception and cognitive processing between healthy students and students with depressive tendencies, which could be potentially explained by the differences of emotional perception through the visual channel between the two groups. For students with depressive tendencies, the impairment in perceiving positive emotion induces the difficulty to further evoke their corresponding positive emotional responses (such as learning motivation), results in the inability to benefit from the positive facial expression of pedagogical agents. In instructional design for students with depressive tendencies, it is important to avoid overreliance on pedagogical agent' positive facial expressions (e.g., sustained smiling, exaggerated eyebrow movements) to arouse learning motivation. The lack of effective positive emotion transmission through facial expressions could be compensated for through intentional visual design (Xie et al., 2019; Van Gog, 2021; Zhang et al., 2024). For example, incorporating visual cues like colors, font size or underline to highlight the key content in multimedia materials. This helps direct students' attention to core information, reduces interference from irrelevant elements, and mitigates cognitive distraction caused by emotional perception bias. On the other hand, it is necessary to control the frequency and intensity of pedagogical agent' negative facial expressions. This prevents reinforcing students' negative attentional bias and reduces cognitive resources.

### 4.2 The impact of emotional expression through the auditory channel

For the auditory channel, this study found that students with depressive tendencies could perceive both positive and negative emotional expression of pedagogical agents. More importantly, compared with pedagogical agent with negative voice tone, positive voice tone significantly enhanced students' social connection with the pedagogical agents, comprising their perception of the agent's role in facilitating learning and its credibility. The result is congruent with healthy groups and support the positivity principle. Moreover, the result also conformed to the emotional contagion theory (Hatfield et al., 1993). The emotional contagion theory points out that an individual's emotional expression would be affected by the emotional expression. Specifically, positive voice tone of the pedagogical agent could promote emotional resonance, which made students have positive emotional responses and promote the accessibility and reliability of the pedagogical agent. This finding also supports Naranjo et al. (2011), the students with depressive tendencies could accurately perceive the positive emotion conveyed by the voice tone, which has the similar pattern with the healthy students.

In terms of specific learning performance, although there were differences in the forms of learning performance between healthy students and students with depressive tendencies, positive voice tone could promote all dimensions of learning. A large number of studies have confirmed that for healthy students, they could keenly perceive emotional cues in voice and respond accordingly (Liew et al., 2017), in detail, positive voice tone not only make students feel close to the pedagogical agent, but also enhances their engagement and learning motivation, and ultimately improves learning performance (Keller et al., 2016; Davis et al., 2019; Lawson and Mayer, 2022). Similarly, for students with depressive tendencies in the present study, although the role of positive voice tone on learning performance was not as obvious as that in healthy groups, positive emotion still effectively promoted the social connection between students and pedagogical agents, specifically, positive voice tone significantly improved students' perception of the pedagogical agent's role in facilitating learning and its credibility. Both groups showed positive feedback to the positive emotional expression of pedagogical agents. This indicates that regardless of whether students have depressive tendencies, the positive voice tone of the pedagogical agent could help them to learn, reflecting the universal applicability of voice tone as an auditory cue for emotional expression in multimedia learning.

The results also showed clearly that students with depressive tendencies had different emotional perception patterns for cues conveyed by visual and auditory channels in multimedia learning. For the visual channel, students showed positive emotional perception impairment, and the positive or negative expression of pedagogical agent had similar effect in the cognitive processing and learning outcomes. However, for the auditory channel, although students had depressive tendencies, they could accurately perceive both positive and negative emotional expression presented by pedagogical agents. The positive voice tone of pedagogical agent significantly enhanced students' social connection with the pedagogical agents, comprising their perception of the agent's role in facilitating learning and its credibility, thereby optimizing their experience in the cognitive process. These results indicate that compare with the visual channel, the auditory channel has a stronger influence on emotional transmission for students with depressive tendencies and could stimulate their positive emotional responses. This might be due to voice tone not only conveys emotional information, but also enhances the intensity of emotional expression through non-verbal characteristics such as intonation and rhythm, making it easier for students to perceive and process (Lawson et al., 2021c; Dinçer, 2022). In addition, we can see the results were inconsistent with the emotional transmission rules in interpersonal communication proposed by Mehrabian (1971), which indicates that the transmission of emotional information in multimedia learning is different from face–to–face communication. In the multimedia learning environment, voice tone, as a kind of accompanying information, could more directly affect students' emotional states, thereby regulating their cognitive processing and learning effects.

To sum up, this study found that the emotion conveyed by voice tone had a more significant impact on the multimedia learning for students with depressive tendencies, which is different from the traditional emotional transmission rules (Mehrabian, 1971). Therefore, when designing multimedia instruction for students with depressive tendencies, auditory emotional cues should be regarded as the priority design element, in order to give full play to their dominant role in emotion transmission and cognitive guidance. For example, when designing the pedagogical agent in instructional videos, priority should be given to using positive voice tone with distinct intonation variations, avoiding monotonous and flat expressions, while taking into account the attention characteristics of students with depressive tendencies, to enhance their emotional perception and learning motivation.

### 4.3 The impact of emotional congruency across visual and auditory channels

The study further analyzed the effect of emotional congruency across dual channels on multimedia learning among students with depressive tendencies. The results revealed that when both visual and auditory channels conveyed positive emotions, students' germane cognitive load increased, which was in line with the positivity principle in multimedia learning (Lawson et al., 2021b; Plass et al., 2020), suggesting that pedagogical agents' positive emotional expression inherently facilitates the learning processing. Notably, the study also discovered that when both channels conveyed negative emotions, students' germane cognitive load also increased, significantly enhancing the transfer test performance. While this outcome contradicts predictions of the positivity principle, it corresponds with the congruency effect (Abramson et al., 2021; Pi et al., 2023).

Specifically, congruent emotional cues across channels help students with depressive tendencies integrate information more effectively, reduce cognitive conflict, and allocate greater cognitive resources to learning materials, thereby improving learning outcomes. The mechanism underlying this effect can be explained by considering the unique cognitive processing characteristics of students with depressive tendencies. These students often exhibit a negative emotion bias and experience difficulties in integrating multimodal information (Joormann and Gotlib, 2010). When emotional cues are congruent (e.g., both channels are negative), the cognitive system does not need to expend extra resources to resolve conflicting signals. This reduction in internal conflict frees up cognitive resources, allowing for more efficient information integration. This streamlined processing ultimately leads to deeper learning and better performance on transfer tests. These findings provide crucial implications for multimedia learning designers. When developing instructional materials for students with depression tendencies, emotional congruency across sensory channels is essential to minimize cognitive conflict, promote synergistic emotional-cognitive processing, and ultimately enhance learning performance.

It is important to note that this study adopted eye-tracking technology to record on-time and objective data on students' cognitive processing, examining attentional allocation patterns and information processing depth during material engagement (Li and Zheng, 2024). Eye tracking technology is widely recognized as an effective tool for objectively and instantaneously reflecting cognitive processes in multimedia learning research (Pi et al., 2023). However, none of the eye-tracking indexes examined in this study revealed significant differences across emotional conditions, sensory channel conditions, or combined dual-channel emotional conditions. This outcome contrasted with the high sensitivity and effectiveness typically demonstrated by eye-tracking methodologies in prior multimedia learning studies. We speculated that the use of a brief instructional video may not adequately capture the emotional complexity of current multimedia learning. Moreover, we speculated that this discrepancy might be intrinsically linked to the distinctive cognitive processing characteristics of participants with depressive tendencies. Individuals with depressive tendencies demonstrate negative attentional bias during information processing, preferentially attending to stimuli matching their emotional state while experiencing difficulties in emotional integration and multimodal information processing (Joormann and Gotlib, 2010). These characteristics impede efficient handling of multiple information streams in complex situations, resulting in slower attentional resource allocation and impaired coordination between informational elements, and ultimately leading to more complex and delayed cognitive processing. Consequently, although participants demonstrated emotion-perception effects in post-learning transfer tests, these processing differences failed to manifest simultaneously in eye-tracking indexes. We emphasized that this interpretation remained speculative, expecting further empirical investigation to explore the cognitive processing patterns of students with depression tendencies.

To our knowledge, this is the first study to adopt eye-tracking technology to investigate multimedia learning among students with depressive tendencies. Future research may utilize this technology for deeper exploration for students with depressive tendencies, with the goals of revealing their unique cognitive processing characteristics and ultimately enhancing learning outcomes for them.

## 5 Limitations and future directions

Several limitations of this study should be emphasized. Firstly, although we conducted the Beck Depression Inventory (BDI; Beck et al., 1961) to diagnose students with depressive tendencies, we did not complete the full Structured Clinical Interview for the DSM-IV Diagnoses (SCID; First et al., 1998). Therefore, it is possible that participants may had other undiagnosed disorders. Secondly, the duration of instructional video was short and may not adequately capture the complexity of authentic multimedia learning environments, thereby constraining ecological validity and limiting the generalizability of the findings. Future research should adopt the longer-duration learning materials, it may be induced by segmented and instructional materials, which are more closely approximate real-world instructional contexts to make learners to adequately capture the complexity of authentic multimedia learning environments. Thirdly, the single topic of the learning materials may also constrain the ecological validity when generalizing findings to other disciplines. Poquet et al. (2018) and Scheiter et al. (2014) mentioned that there is a critical need to expand research on video-based learning to incorporate a broader range of disciplines (e.g., medicine, engineering and STEM) and difficulty levels (e.g., introductory vs. advanced courses). Furthermore, Mayer (2024) also suggested that future research should explore interdisciplinary applications (e.g., combining physics simulations with narrative storytelling) and differential difficulty levels (e.g., adaptive multimedia systems that adjust content complexity in real time). Therefore, future research may incorporate a broader range of topics, spanning diverse disciplines and difficulty levels, to comprehensively test the generalizability of current conclusions. Fourthly, this study adopted eye-tracking technology to record learners' cognitive processing, but none of the eye-tracking indexes revealed significant differences across different conditions, further eye-tracking investigations are still needed for students with depression tendencies. Future research may also adopt multidimensional indexes and psychophysiological tools (e.g., EEG, fNIRS, fMRI) to explore learners' cognitive processing for students with depression tendencies.

## 6 Conclusions

(1) When emotional cues conveyed by visual channel, students with depressive tendencies could perceive the negative emotional expression but could not effectively perceive the positive ones, supporting Beck's cognitive theory of depression.
(2) Emotional cues conveyed by auditory channel significantly influenced emotional perception and cognitive processing in students with depressive tendencies, providing evidence for both the positivity principle and emotional contagion theory.
(3) Emotionally congruent dual-channel cues facilitated multimedia learning performance in students with depressive tendencies, evidencing the congruency effect.

## Data Availability

The datasets presented in this article are not readily available because the raw data supporting the conclusions of this article will be made available by the authors, without undue reservation. Requests to access the datasets should be directed to cuilei_cn@163.com.
